# Towards assessing and improving the reliability of ultrashort echo time quantitative magnetization transfer (UTE-qMT) MRI of cortical bone: In silico and ex vivo study

**DOI:** 10.1007/s10334-024-01190-7

**Published:** 2024-08-10

**Authors:** Soo Hyun Shin, Dina Moazamian, Qingbo Tang, Saeed Jerban, Yajun Ma, Jiang Du, Eric Y. Chang

**Affiliations:** 1 Department of Radiology, University of California San Diego, 9452 Medical Center Drive, La Jolla, CA USA; 2 Radiology Service, Veterans Affairs San Diego Healthcare System, 3350 La Jolla Village Drive, San Diego, CA USA; 3 Shu Chien-Gene Lay Department of Bioengineering, University of California San Diego, La Jolla, CA USA

**Keywords:** Ultrashort echo time (UTE), Quantitative magnetization transfer (qMT), MRI, Cortical bone, Digital phantom

## Abstract

**Objective:**

To assess and improve the reliability of the ultrashort echo time quantitative magnetization transfer (UTE-qMT) modeling of the cortical bone.

**Materials and Methods:**

Simulation-based digital phantoms were created that mimic the UTE-qMT properties of cortical bones. A wide range of SNR from 25 to 200 was simulated by adding different levels of noise to the synthesized MT-weighted images to assess the effect of SNR on UTE-qMT fitting results. Tensor-based denoising algorithm was applied to improve the fitting results. These results from digital phantom studies were validated via ex vivo rat leg bone scans.

**Results:**

The selection of initial points for nonlinear fitting and the number of data points tested for qMT analysis have minimal effect on the fitting result. Magnetization exchange rate measurements are highly dependent on the SNR of raw images, which can be substantially improved with an appropriate denoising algorithm that gives similar fitting results from the raw images with an 8-fold higher SNR.

**Discussion:**

The digital phantom approach enables the assessment of the reliability of bone UTE-qMT fitting by providing the known ground truth. These findings can be utilized for optimizing the data acquisition and analysis pipeline for UTE-qMT imaging of cortical bones.

**Supplementary Information:**

The online version contains supplementary material available at 10.1007/s10334-024-01190-7.

## Introduction

Bone fractures are a growing public health issue posing a serious worldwide healthcare and economic burden [1]. The risk of bone fractures is significantly increased in people with osteoporosis and diabetes, with the global prevalence of each disease estimated to be 19.7% [2] and 10.5% [3], respectively. The most widely used fracture risk assessment is dual-energy X-ray absorptiometry (DXA)-based bone mineral density (BMD) measurement. However, clinical studies have reported that the BMD measurement only explains 30–50% of fractures [4–6]. This limited sensitivity of BMD has motivated the need for more reliable fracture risk assessment tools that focus on not only the BMD but also other features and constituents of the bone, such as bone microstructure [7] and organic matrix [8, 9].

Magnetic resonance imaging (MRI) can not only provide anatomical images but also quantitative information on molecular components of tissues, leveraging its numerous contrast mechanisms. Quantitative magnetization transfer (qMT) imaging is one of the widely studied MRI methods for probing the macromolecular content and their properties in tissues [10–12]. While the usage of qMT imaging has been limited to soft tissues [13–17] (e.g., brain, muscle, spinal cord and kidney) due to the very short T_2_* relaxation of hard tissues, combining qMT with an ultrashort echo time (UTE-qMT) readout sequence has enabled the application of qMT analysis to measure the macromolecular fraction (MMF) of cortical bones [18–20]. Other qMT parameters such as magnetization exchange rates between the free water and macromolecular pools may also provide insights into the quality of the bone that accounts for fracture risk [21, 22].

Yet, even with the use of UTE readouts, it is unclear whether the signal-to-noise ratios (SNR) of bone MT-weighted images is sufficient for qMT modeling. From previous MT studies on other tissues, it is known that the measurements of exchange rates are highly affected by the SNR of the images, and to a much greater degree than the MMF measurements are [23, 24]. Such SNR-based reliability of qMT modeling has not been studied for UTE-qMT imaging of bones. Towards the goal of developing qMT parameters as robust imaging markers that are correlated to bone fracture risk, a systematic assessment of the robustness and reliability of bone UTE-qMT must be performed (including the minimum SNR requirements) and strategies must be established for improving qMT parameter measurements.

In this study, we built a digital phantom through UTE-qMT simulation that mimics the MR properties of cortical bones. Unlike in vivo or ex vivo qMT studies, the simulation-based approach allows one to examine whether the qMT fitting is robust and reliable as the ground truths are known. Multiple series of MT-weighted images were synthesized to generate a wide range of SNR levels to examine the effect of SNR on qMT fitting results. The number of data points and the initial points for the qMT fitting were controlled to simulate how acquisition and analysis pipelines can affect the qMT fitting result. An ex vivo rat leg bone was scanned to validate the digital phantom results. For both digital phantom and ex vivo data, a tensor-based multidimensional denoising algorithm [25] was tested as a potential solution for the inherently low SNR of bone MRI and compared its performance with conventional Gaussian filtering.

## Materials and methods

### Digital phantom preparation

**Fig. 1:**
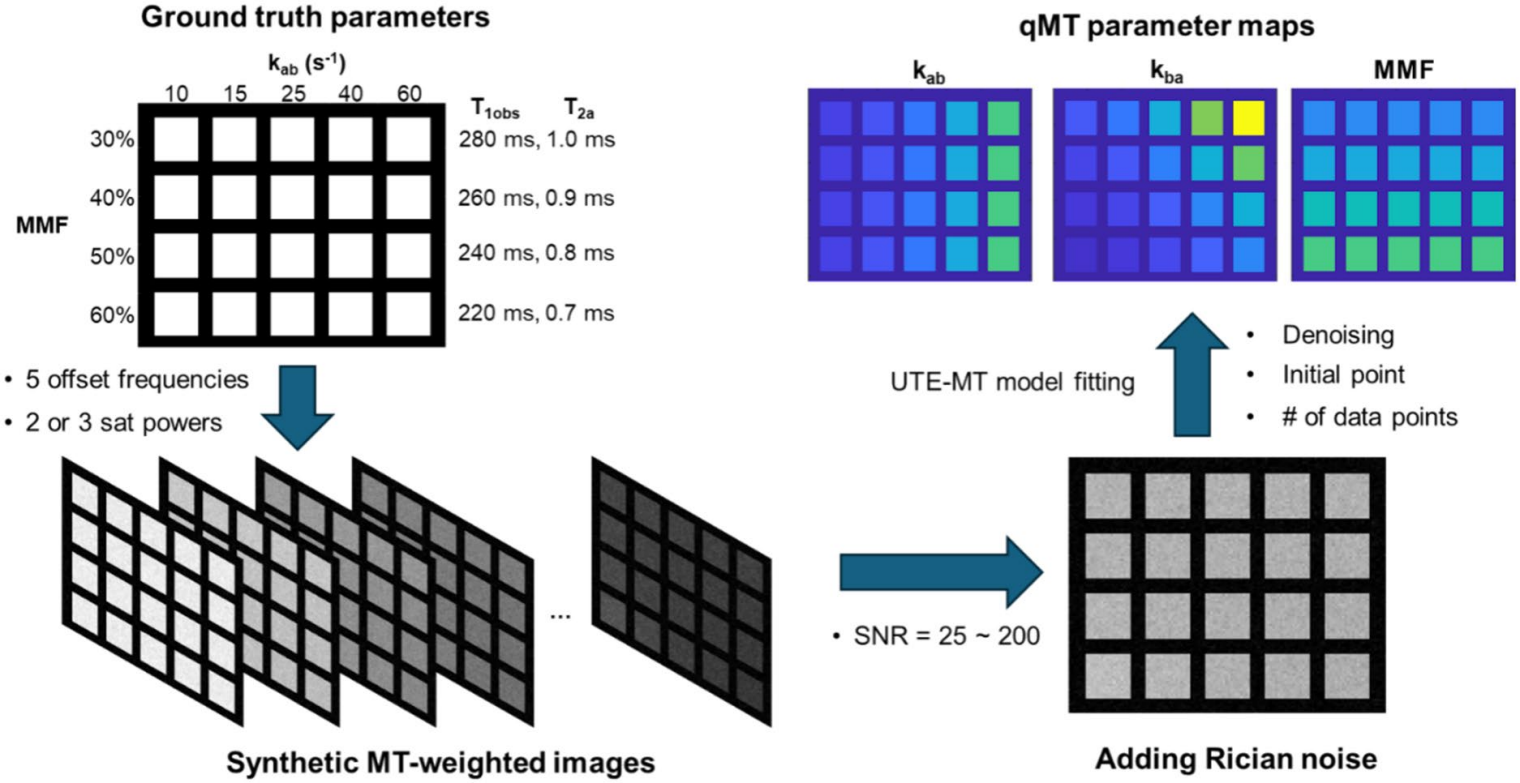
Schematic flow of digital phantom preparation and qMT fitting. Twenty digital cortical bone chip images acquired at two or three saturation powers and five offset frequencies are simulated based on the UTE-qMT parameters measured from previous cortical bone studies. The Rician noise is added to the simulated bone chip images to generate the range of SNR levels from 25 to 200. The simulated bone chip images with added noise are fitted back to the UTE-qMT model to measure the macromolecular fraction (MMF) and exchange rates (*k*_ab_ and *k*_ba_) by testing a different number of data points, selection of initial points of nonlinear fitting and applying a denoising algorithm

Digital phantom preparation was performed using custom-written MATLAB code (MathWorks, Natick, MA). The overall flow of phantom preparation and analysis is summarized in Fig. 1.

The binary spin bath (BSB) model was assumed for the digital phantom generation, modeling the cortical bone as a combination of two compartments, the free water pool (pool *a*) and the macromolecular pool (pool *b*) [26]. The BSB model with a pulsed saturation scheme can be well described by the rectangular pulse (RP) model by Sled and Pike [27], which was further modified for multiple acquisitions after a single MT preparation pulse [21]. This modified RP model was used for both generating digital phantoms and subsequent fitting for testing different SNR levels, the number of data points, and the effect of initial points for nonlinear fitting.

The modified RP model is described by a total of seven parameters: The size of the free water pool (M_0a_), longitudinal relaxation rates of the free water and macromolecular pool (R_1a_ and R_1b_), exchange rate from free water to macromolecular pool (k_ab_), MMF (defined as M_0b_/(M_0a_ + M_0b_)), T_2_ of free water and macromolecular pools (T_2a_ and T_2b_). As shown in previous studies, R_1b_ can be fixed to 1 s^−1^ [26, 28–30]. R_1a_ can be determined by other parameters and observed T_1_ (e.g., T_1obs_ = 1/R_1obs_):

$${R}_{1a}= {R}_{1obs}- \frac{{k}_{ab}({R}_{1b}-{R}_{1obs})}{{R}_{1b}- {R}_{1obs}+\frac{{k}_{ab}(1-MMF)}{MMF}}$$

Thus, with the *R*_1obs_ measured, a total of five parameters can be determined through fitting. The exchange rate from the macromolecular pool to the free water pool (k_ba_) can be determined as ${k}_{ab}\frac{1-MMF}{MMF}$

The input qMT parameters for generating digital phantoms were chosen from previous studies on UTE-qMT imaging of cortical bones [21, 31] (MMF = 30–60%, *k*_ab_ = 10–60 s^−1^, *T*_1obs_ = 220–280 ms, *T*_2a_ = 0.7–1.0 ms, *T*_2b_ = fixed to 15 µs [32]). Twenty combinations of these parameters were used for simulating different conditions of cortical bones. For each condition, 324 qMT spectra were simulated to create a digital bone chip with the size of 18 × 18 voxels. Three MT saturation powers (3SP, flip angle = 400˚, 800˚, 1200˚) and five offset frequencies (2, 5, 10, 20, 50 kHz) were used to generate qMT spectra. To simulate different levels of SNR, Rician noise with zero mean and different levels of standard deviation were added to the images so that the SNR of the image at the lowest saturation power (400˚) and the largest offset frequency (50 kHz) ranges from 25 to 200. SNR was calculated as the mean signal intensity of digital phantoms divided by the standard deviation of the Rician noise used for noise generation. All the SNRs reported in this study are based on the image simulated or acquired at the lowest saturation power and the largest offset frequency unless indicated otherwise.

### Digital phantom analysis

The prepared digital phantom images were fitted back into the UTE-qMT model to quantify the MMF and exchange rates (*k*_ab_ and *k*_ba_) and observe whether the results matched the input parameters used for generating the phantoms. The non-linear fitting was performed using the ‘lsqcurvefit’ function in MATLAB with the default Trust-region fitting algorithm.

To assess the effect of the number of saturation powers and offset frequencies, we tested the full set of MT spectra (3 saturation powers and 5 offset frequencies, 15 data points) and the MT spectra with only 2 saturation powers (2SP, 800˚ dataset excluded, 10 data points). For this assessment, the initial point of non-linear fitting was fixed to the ground truth of each phantom so that only the effect of the number of data points can be assessed. The effect of the initial point of the nonlinear fitting process was also tested by using the initial point of either the ground truth of each phantom or the fixed one (MMF = 50%, *k*_ab_ = 25 s^−1^, *T*_1obs_ = 240 ms, *T*_2a_ = 0.8 ms), near the midpoint of the range of parameters tested. All these assessments were done at different SNR levels.

A denoising algorithm was tested on the digital phantoms to examine whether qMT parameter fitting results improved. Among numerous potential algorithms, we tested a recently developed tensor Marchenko-Pastur distribution Principal Component Analysis (tMPPCA) method [25]. The digital phantom image with the SNR of 50 was denoised by tMPPCA with a window size of 3 × 3 × 3 [33]. The Gaussian filtering with the kernel standard deviation fixed to 1 was also tested for comparison.

### Ex vivo* rat bone MR image acquisition and analysis*

An ex vivo rat leg bone was scanned on a 3 T scanner (Bruker, Billerica, MA) with a 1 cm loop coil to confirm the digital phantom results. The bone marrow of the bone was removed and placed in Fomblin (Ausimont, Thorofare, NJ) for susceptibility-matching purposes. MT-weighted UTE images were acquired at three saturation powers (500˚, 1000˚, 1500˚) and five offset frequencies (2, 5, 10, 20, 50, kHz). Readout parameters are as follows: TR/TE = 86/0.026 ms, number of spokes per MT saturation = 13, inter-spoke TR = 5 ms, flip angle = 10˚, field-of-view (FOV) = 10 mm × 10 mm × 80 mm, matrix = 84 × 84 × 84, receiver bandwidth = 100 kHz. The MT-weighted image acquisition was repeated 64 times to manually control the number of averages (NA). NA of 1, 4, 8, 16, 32, and 64 were tested to match with the SNR of 25–200 used for digital phantom simulation. For the qMT fitting process, the *T*_1obs_ was assumed to be 250 ms [31, 34–38]. The effect of denoising on MT parameter fitting was also tested using the same tMPPCA denoising algorithm. All the analysis was performed twice, once with the full dataset (3SP) and once with 2 saturation powers (2SP, 1000˚ dataset excluded).

## Results

**Fig. 2:**
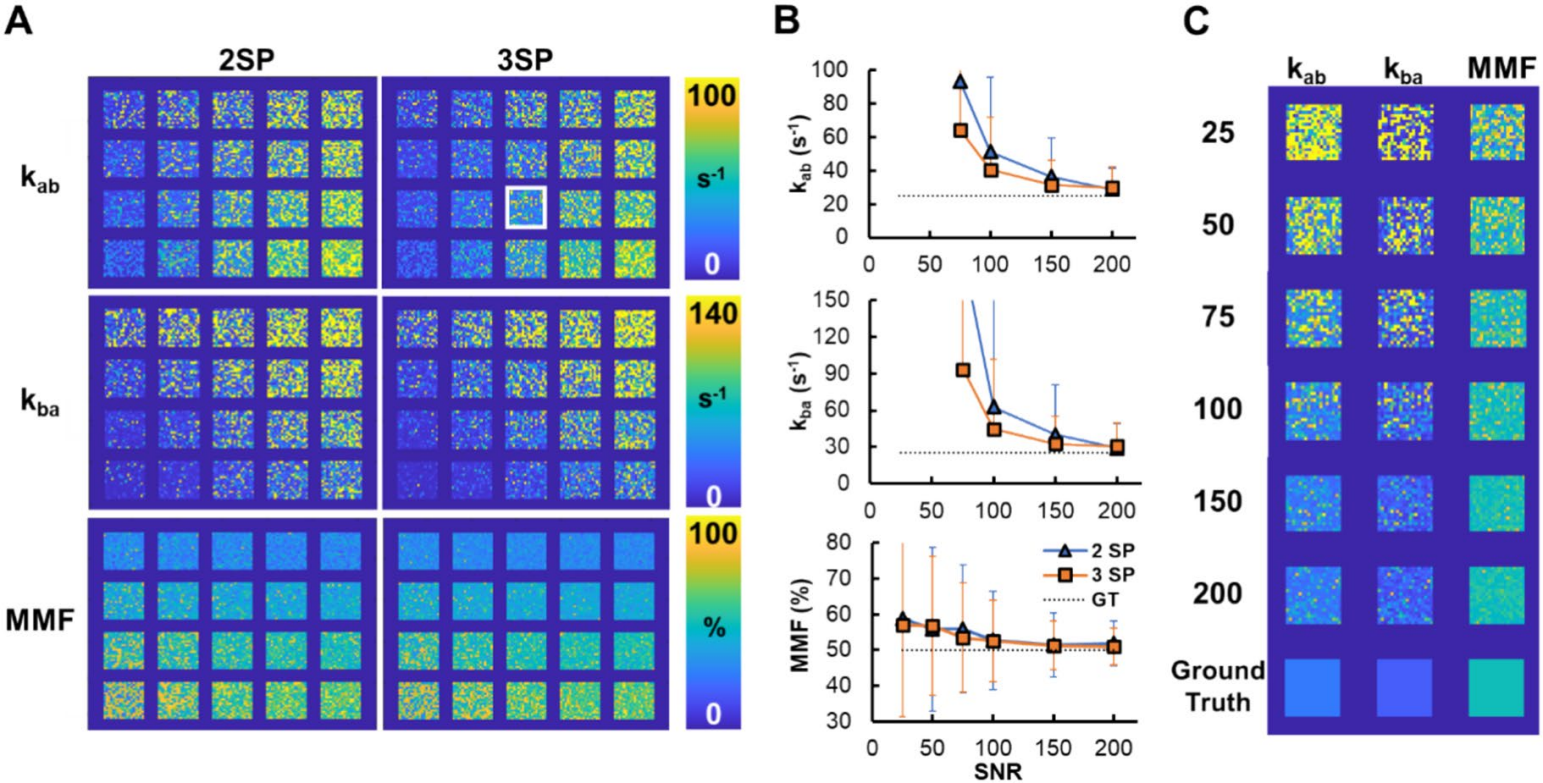
The effect of the number of data points and signal-to-noise ratio (SNR) on cortical bone UTE-qMT fitting. The UTE-qMT parameters acquired via fitting 2 saturation powers (400˚ and 1200˚, 2SP) and 3 saturation powers (400˚, 800˚ and 1200˚, 3SP) are compared. **A** UTE-qMT parameter maps generated from the dataset with SNR of 100 with the initial point chosen as the ground truth of each phantom. **B** The exchange rates (*k*_ab_ and *k*_ab_) show the fitting results are more stable and closer to the ground truth upon using the 3SP dataset. Macromolecular fraction (MMF) measurements are stable for both 2SP and 3SP datasets. The 2SP dataset shows higher spatial variation of parameter fitting represented as larger standard deviations of the measurements. The measurements are from the phantom with the white box shown in A. *k*_ab_ and *k*_ba_ measurements are only shown at SNR of 75 or above due to abnormally large values from unstable fitting. **C** UTE-qMT parameters maps of the phantom analyzed (white box in A) at different SNR levels (25–200) and the maps of ground truth parameters

Digital phantom simulation shows similar MMF fitting results from MT spectra with 2SP and 3SP datasets (Fig. 2A), whereas the exchange rate measurements are slightly improved on the 3SP dataset. The MMF measurement is relatively robust throughout the SNR levels tested ranging from 58.8 ± 27.4% (2SP) and 57.0 ± 25.7% (3SP) at SNR of 25 to 51.8 ± 6.3% (2SP) and 51.0 ± 5.1% (3SP) at SNR of 200 (Fig. 2B). The exchange rate measurements are unstable in the lower SNR levels and become comparable to the ground truth at the SNR of 150 or above. These trends are also shown in the parameter maps at different SNR levels shown in Fig. 2C.

**Fig. 3:**
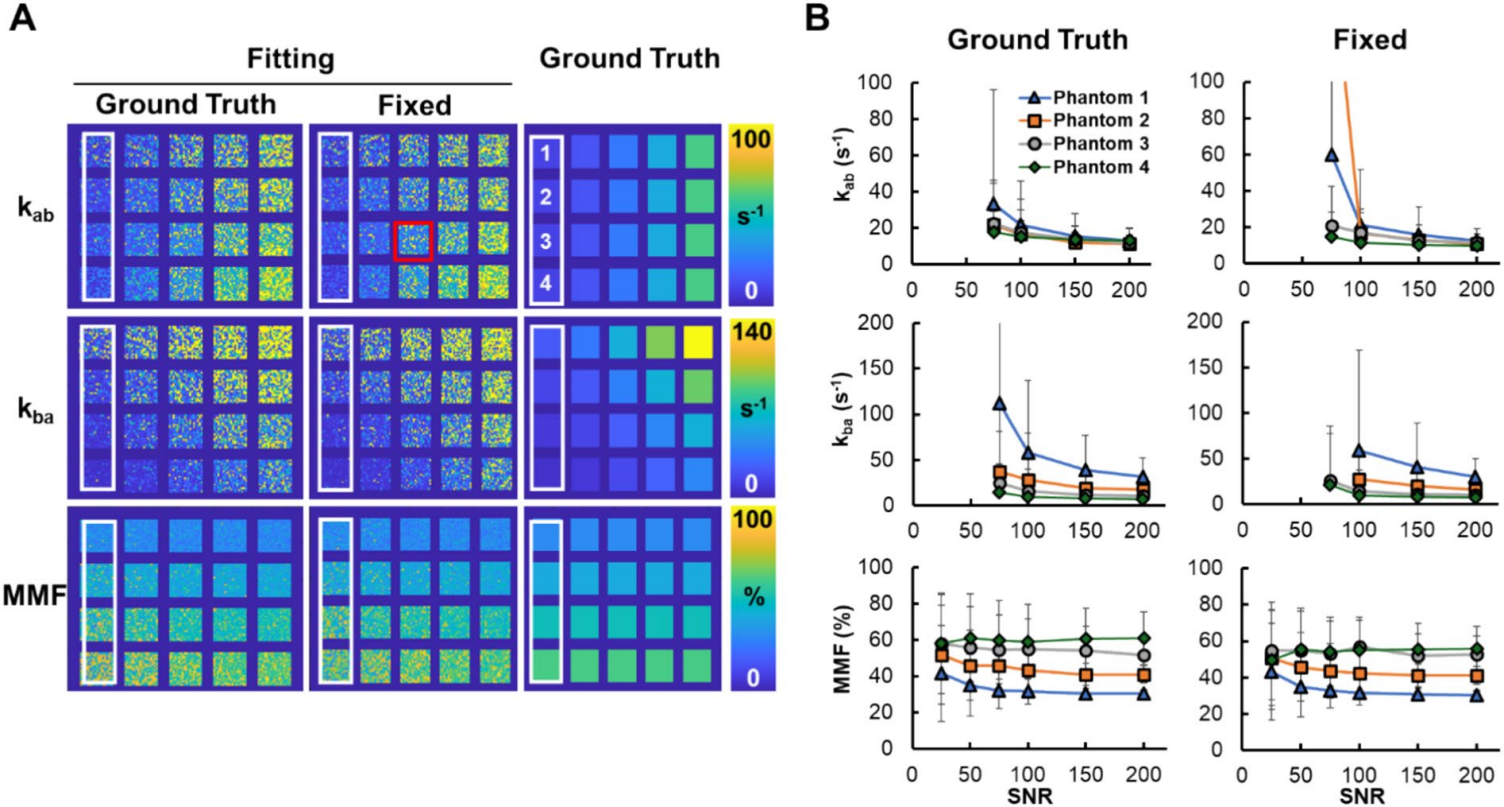
The effect of the choice of initial point of nonlinear fitting of the UTE-qMT model. **A** UTE-qMT parameter maps from digital phantoms with three saturation powers and an SNR of 100. Parameter maps in the first two columns (Fitting) are the fitting results from using the initial point of either the ground truth of each phantom (Ground Truth) or the fixed values (parameters used for the phantom with the red box; Fixed). Ground truth maps are also included in the right-most column for comparison. **B** The macromolecular fraction (MMF) and exchange rate (*k*_ab_ and *k*_ba_) measurements from 4 phantoms indicated in the white boxes shown in A. *k*_ab_ and *k*_ba_ measurements are only shown at SNR of 75 or above due to abnormally large values from unstable fitting

Similar trends are seen from the assessment of the initial point effect (Fig. 3). Overall, the choice of initial points did not significantly affect the fitting results, while the MMF measurement is more stable than the exchange rate measurements (Fig. 3A). Exchange rate measurements are reliable at the SNR of 150 or above, regardless of the choice of the initial point for nonlinear fitting (Fig. 3B). Still, the MMF measurement shows that the initial point set to the ground truth gives a more accurate result than the fixed initial point for certain phantoms at high SNR levels. This better measurement of MMF is also translated to a marginally improved measurement of k_ba_ in certain phantoms (Fig. 3).

**Fig. 4:**
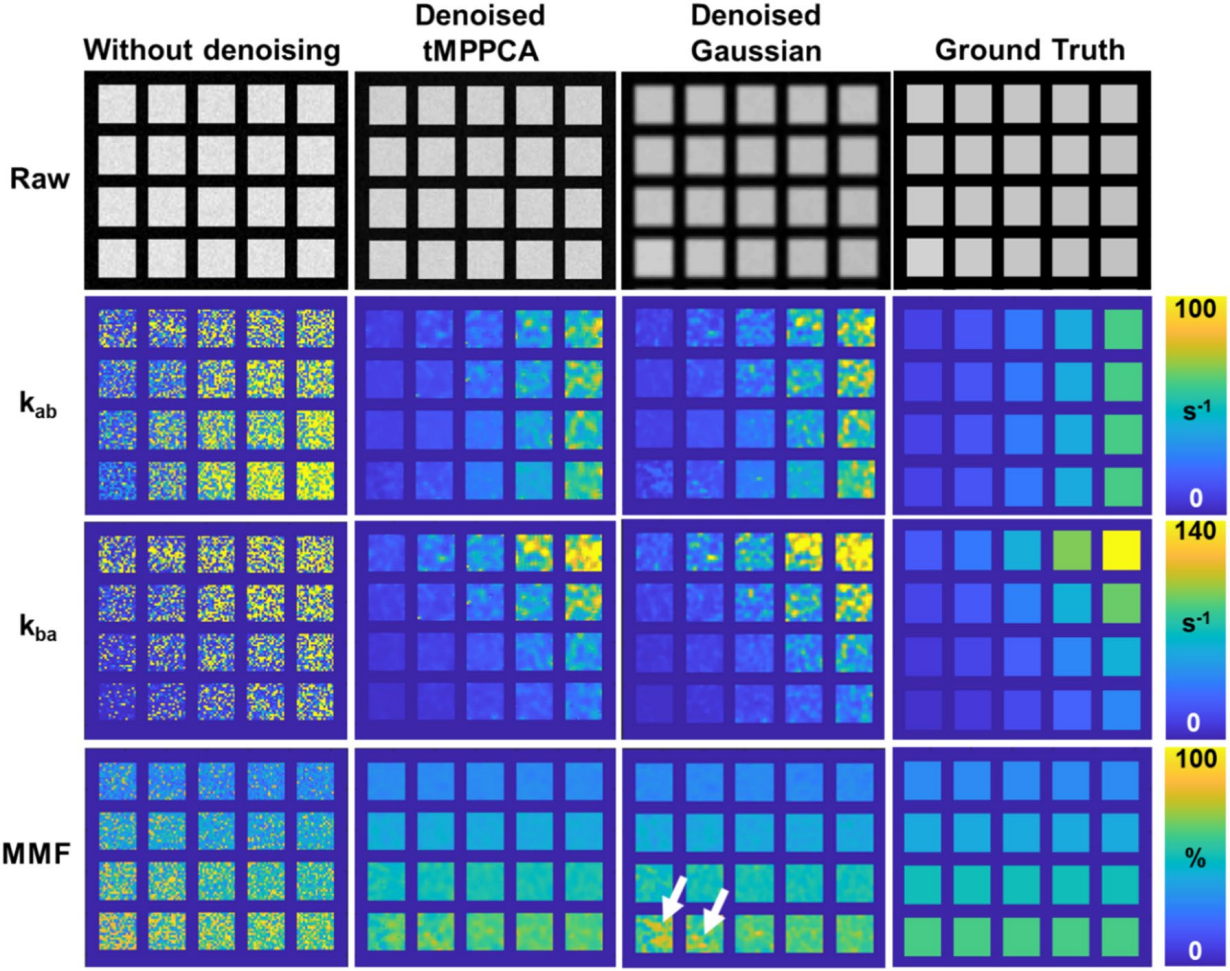
tMPPCA denoising improves the UTE-qMT fitting of simulated digital phantoms. The simulated digital phantom with three saturation powers and an SNR of 50 was tested. The initial point of fitting was selected as the ground truth of each phantom. Both tMPPCA and Gaussian denoising have significantly improved the UTE-qMT parameter fitting with less noise in the parameter map, and the results are more comparable to the ground truth. The white arrows indicate the difference in MMF measurement between tMPPCA and Gaussian denoising

The improvement of qMT fitting via denoising was also observed (Fig. 4). Although following the patterns in the ground truth, the exchange rate maps are highly noisy due to the unstable fitting without denoising. Both tMPPCA and Gaussian denoising of the raw digital phantom images generate the parameter maps that are closer to the ground truths, albeit with some residual regional variations in the parameter maps. Interestingly, the tMPPCA algorithm showed a more accurate measurement of high MMF values than the Gaussian filtering.

**Fig. 5:**
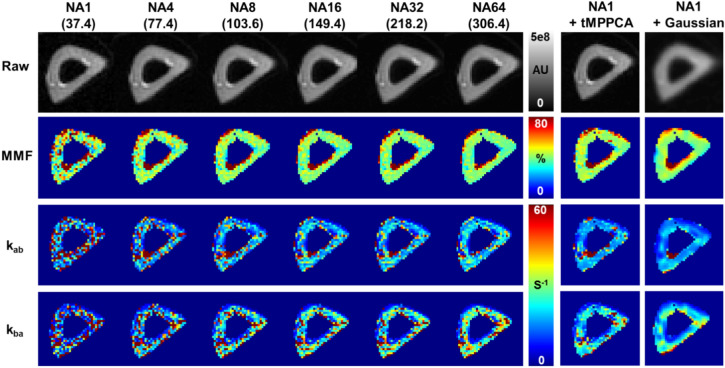
Ex vivo rat leg bone scans with different degrees of averaging. The number of averages (NA) was controlled from 1 to 64 to cover the SNR range of 25–200 simulated for digital phantoms. The SNR levels are indicated in parentheses. Denoising via the tMPPCA algorithm and Gaussian filter were also tested on the NA1 dataset (NA1 + dn). The macromolecular fraction (MMF) fitting is stable except for the one from the NA1 dataset, whereas the exchange rate maps (*k*_ab_ and *k*_ba_) are steadily improving with the increasing NA. The tMPPCA-denoised NA1 dataset shows comparable results with the NA32 and NA64 datasets. The denoising with the Gaussian filter also shows improved exchange rate measurement along with a significant blurring in both raw image and parametric maps. The UTE-qMT parameter maps shown here are generated using the full dataset acquired (three saturation powers)

To validate the digital phantom results, ex vivo rat bones were scanned, and the improvement of qMT fitting was observed along with increasing the number of averages (Fig. 5). The SNR of raw images (saturation power = 500˚, offset frequency = 50 kHz) increased from 37.4 (NA = 1) to 306.4 (NA = 64), matching with the SNR levels tested in the digital phantom simulations. Denoising via the tMPPCA method was tested on the NA = 1 dataset, which showed substantial improvement of SNR (131.6) and the subsequent qMT fitting that generated a result comparable to the one from the NA = 64 dataset with preserved spatial resolution (Fig. 5). The Gaussian filtering also showed improvement in parameter fitting, but substantial spatial blurring is also observed as expected.

**Fig. 6:**
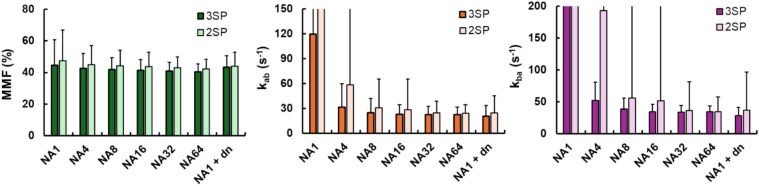
Ex vivo rat bone scan measurements. The UTE-qMT fitting results shown in Fig. 5 were measured with a region of interest covering the entire rat bone. Macromolecular fraction (MMF) measurements show stable measurements across the range of number of averages (NA) used, regardless of using 2 saturation powers (2SP) and 3 saturation powers (3SP). The measurement of exchange rates (*k*_ab_ and *k*_ba_) is more NA-dependent than MMF measurements. The variation of exchange rate measurements is also significantly reduced when using the 3SP dataset. Due to significant spatial blurring upon Gaussian denoising, only the results from tMPPCA denoising (NA1 + dn) are shown

The quantitative measurements of qMT parameters from ex vivo scans also show the robust measurement MMF across all NAs tested, whereas the exchange rate measurements are not reliable at NA of 1 (Fig. 6). Compared to digital phantoms, however, low SNR ex vivo scans (NA = 1, 4, 8) show much more comparable results to those from high SNR scans (NA = 32 and 64). The tMPPCA denoising of the dataset with NA of 1 substantially improved the quality of fitting compared to the original image. Despite the overestimated MMF (43.4 ± 7.2 vs. 40.4 ± 5.2%; Fig. 6A) and underestimated exchange rates (*k*_ab_: 20.7 ± 12.8 s^−1^ vs. 22.6 ± 9.1; *k*_ba_: 28.2 ± 21.2 s^−1^ vs 34.2 ± 18.6 s^−1^; Fig. 6B, C), the results from the denoised NA = 1 dataset are comparable to those from the NA = 64 dataset. The use of the 3SP dataset significantly reduced the variation of all the UTE-qMT parameter measurements in the given ROI compared to the results from the 2SP dataset.

## Discussion

Here, we systematically assessed the SNR requirements and how the fitting process affects the UTE-qMT imaging of bone. Similar to qMT imaging of other tissues, the MMF measurement was robust across a range of SNR levels, whereas exchange rate measurements became accurate when the SNR reached around 150 or above. The UTE-qMT fitting process also turned out to be robust against the selection of the initial point of the nonlinear fitting process, and the 2SP dataset generated comparable results as those from 3SP datasets. The denoising algorithm tested in this study substantially improved the fitting accuracy. These results were also reproduced in ex vivo rat leg bone scans, with relatively more robust MMF measurements and substantial improvement of qMT measurements after denoising.

The advantage of the digital phantom approach demonstrated in this study is the known ground truth. Although the quality of qMT fitting is usually measured by the goodness-of-fit, whether the ground truth value is obtained through the fitting cannot be known by actual scans, unless followed by validation studies such as histology or biochemical assays from tissue samples. By knowing the ground truth values, the digital phantom simulation approach allows examining whether the qMT fitting provides correct results and permits subsequent optimization of the data acquisition and analysis pipeline. Optimizing the qMT fitting pipeline using the known ground truth is also expected to facilitate the development of more advanced techniques for qMT fitting such as neural network-based approaches by providing more accurate and refined training datasets [39, 40].

Comparing the fitting results with the ground truth showed that the exchange rate measurements are highly SNR dependent whereas MMF measurements are more robust even in the low SNR regime, regardless of the number of data points and initial points tested. The unstable fitting of exchange rates at low SNR has been previously shown from qMT studies on other soft tissues [23, 41]. The qMT study on human patellar cartilage reported that the percentage change of the exchange rate becomes lower than 1% only after the SNR of an image becomes 75 or higher [23]. Our digital phantom-based analysis shows that at least an SNR of 100–150 is needed for reasonable voxel-based measurements of exchange rates, whereas MMF measurements are acceptable even with an SNR of around 50. The unstable fitting of exchange rates at SNR of 50 or lower generated unrealistically large values (k_ab_ > 10^3^ and k_ba_ > 5 × 10^3^ s^−1^) and variations that had to be excluded from the measurements (Figs. 2B and 3B). The instability of exchange rate measurements may be alleviated by setting up narrower but still realistic boundary conditions during the fitting process. Still, considering the inherently low SNR of actual bone MR images acquired in a clinically feasible scan time, only a region-of-interest (ROI)-based analysis seems applicable for the exchange rate measurements unless certain strategies to improve the SNR are employed, such as low-pass filtering and other denoising algorithms.

As a potential method of improving the SNR and corresponding qMT fitting results, we tested the tMPPCA algorithm on both digital phantoms and rat bone data and compared it with conventional Gaussian filtering. The tMPPCA is designed for denoising multidimensional MRI data by leveraging the redundancy in the extra dimensions [25]. This algorithm has demonstrated substantial improvement of SNR of multi-echo images, diffusion-weighted images, and T_1_-weighted images, as well as subsequent parameter fitting results [25, 33]. We hypothesized that this algorithm would also bring high SNR gain to qMT datasets as qMT data are inherently multidimensional due to repeated acquisitions at different saturation powers and offset frequencies. The digital phantom simulation showed that tMPPCA denoising enables accurate voxel-wise measurement of exchange rates even with an SNR of 50, similar to the results from Gaussian filtering. This result was validated by applying the same algorithm to the rat bone dataset, shown by the similar fitting results between the raw high SNR dataset (NA = 64) and the denoised low SNR dataset (NA = 1). Compared to Gaussian filtering, the tMPPCA denoising did not show any spatial blurring. These results indicate that even with the low SNR raw data, the exchange rates can also be reliably measured with a proper denoising strategy.

While MMF alone can be a great imaging marker of the content of organic matrix in the bone, exchange rates can also be valuable markers of macromolecule conditions in the bone. With the growing evidence that BMD is not sufficient to examine the bone fracture risk, bone quality is drawing more attention as another determinant of bone fracture risk [42–44]. For instance, non-enzymatic crosslinking of collagen fibrils via advanced glycation end products in the cortical bone is considered to be a key contributor to the increased fracture risk in type 2 diabetes patients despite the preserved or even elevated BMD measurements [45–47]. Since the exchange rate measurements were demonstrated to be altered upon crosslinking collagens in cornea and cartilage, as well as other polymers [48–50], robust measurements of exchange rates via UTE-qMT modeling can potentially be a valuable marker of assessing the bone quality and fracture risk.

A typical limitation of qMT parameter measurements via the BSB model is parameter correlation. Previous studies have shown that MMF and k_ab_ measurements can be coupled, rather than independent [24, 51]. This parameter coupling was also observed in this study, as shown in the scatter plot of voxel-wise measurement of MMF and k_ab_ (Figure S1). An anisotropic distribution of MMF and k_ab_ measurements was observed throughout the SNR levels tested, indicating that these parameter measurements are not independent. The overall trend of MMF and *k*_ab_ measurement shows the tendency to compensate for the underestimation of MMF with the overestimation of *k*_ab_ and vice versa, which is a previously reported phenomenon [27]. Other qMT parameter estimation approaches that do not depend on nonlinear fitting, such as the dictionary-matching method [52], may alleviate the issue.

In this study, we only tested the effect of SNR, the number of data points, and the selection of initial points for the fitting, but other parameters involved in the image acquisition and analysis procedure can also be tested using the digital phantom approach in the future. The number and selection of offset frequencies and saturation powers can be further tested to identify the combination of these parameters that gives the best fitting accuracy with the minimum data acquisition to reduce the scan time. Testing for the effect of B_1_ inhomogeneity and T_1_ relaxation time is another validation test that may be performed through digital phantoms. In this study, we assumed a perfect B_1_ homogeneity and T_1_ relaxation time measurement. For in vivo UTE-qMT scans of cortical bone, however, the accurate measurements of B_1_ inhomogeneity and T_1_ relaxation are challenging due to the short T_2_* of the cortical bone [34]. Examining the tolerance of B_1_ and T_1_ errors during the qMT fitting will give a better assessment of the reliability of the UTE-qMT imaging. In that regard, the ex vivo rat bone scan results can be improved with the actual measurement of B_1_ inhomogeneity and T_1_. These studies should also be validated through in vivo scans in the future. Due to the higher body temperature and other tissues surrounding the cortical bone (e.g., bone marrow and muscle), the SNR of in vivo bone scan is expected to be lower than the ex vivo scans [53]. The digital phantom approach taken in this study may also be biased due to the discrepancy between the in vivo conditions and the model chosen for generating digital phantoms and subsequent fitting. Whether the findings through the digital phantom demonstrated in this study can be applied to in vivo scans should be further examined. Finally, we chose the tMPPCA algorithm with a fixed window size for denoising. Other denoising algorithms, as well as image filtering and ROI-averaging with different numbers of voxels within an ROI, can also be tested for establishing a robust UTE-qMT analysis pipeline.

## Conclusion

Here, we demonstrated the usage of digital phantom simulation to assess the reliability of qMT measurements. Similar to qMT imaging of other tissues, cortical bone digital phantoms showed robust MMF measurements whereas exchange rate measurements were unstable in low SNR levels. The number of data points and the selection of initial points tested in this study yielded negligible effects on the UTE-qMT fitting results. Denoising via the tMPPCA method showed substantial improvement in qMT fitting in both simulation and ex vivo scans, supporting the feasibility of reliable voxel-wise measurements of bone UTE-qMT parameters.

## Supplementary Information

Below is the link to the electronic supplementary material.

Supplementary file1 (DOCX 57 KB)

## Data Availability

The data supporting the reported findings and the code used for analyzing the data are available from the corresponding authors upon reasonable request.
