# Supplementary material for: Towards assessing and improving the reliability of ultrashort echo time quantitative magnetization transfer (UTE-qMT) MRI of cortical bone: In silico and ex vivo study
Source: MAGMA. 2024 Aug 10;37(6):983–92. doi: 10.1007/s10334-024-01190-7 (PMC11582156; doi:10.1007/s10334-024-01190-7)
Supplement: Supplementary file 1 — Supplementary file1 (DOCX 57 KB) [file 10334_2024_1190_MOESM1_ESM.docx]

**Supplementary Figure**


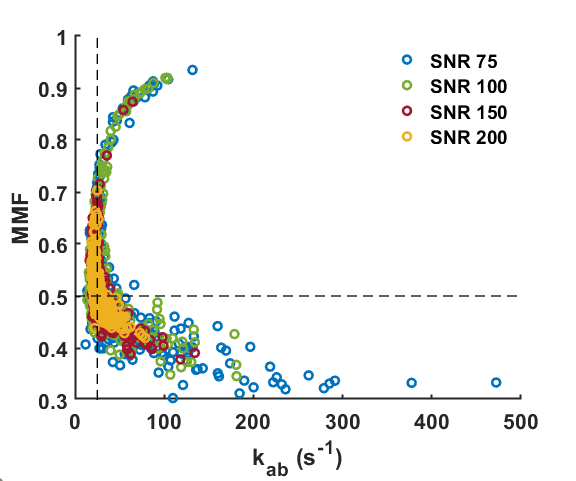


**Figure S1**. A scatter plot of voxel-wise measurement of macromolecular fraction (MMF) and exchange rate (k_ab_). The 324 measurements are from the digital phantom with MMF = 50% and k_ab_ = 25 s^-1^, as indicated by the dashed lines. The distribution of MMF and k_ab_ measurements becomes closer to the ground truth as the SNR increases. The anisotropic distribution indicates the correlation between MMF and k_ab_ measurements, compensating for the underestimation of one parameter by the underestimation of the other one.
